# Improving physiological relevance of cell culture: the possibilities, considerations, and future directions of the ex vivo coculture model

**DOI:** 10.1152/ajpcell.00473.2022

**Published:** 2022-12-26

**Authors:** Sophie L. Allen, Bradley T. Elliott, Brian P. Carson, Leigh Breen

**Affiliations:** ^1^School of Sport Exercise and Rehabilitation Sciences, University of Birmingham, Birmingham, United Kingdom; ^2^NIHR Birmingham Biomedical Research Centre, University Hospitals Birmingham NHS Foundation Trust, University of Birmingham, Birmingham, United Kingdom; ^3^Translational Physiology Research Group, School of Life Sciences, University of Westminster, London, United Kingdom; ^4^Department of Physical Education and Sport Sciences, Faculty of Education and Health Sciences, University of Limerick, Limerick, Ireland; ^5^Health Research Institute, University of Limerick, Limerick, Ireland; ^6^MRC-Versus Arthritis Centre for Musculoskeletal Aging Research, University of Birmingham, Birmingham, United Kingdom

**Keywords:** cell culture, ex vivo, in vitro, plasma, serum

## Abstract

In vitro models provide an important platform for the investigation of cellular growth and atrophy to inform, or extend mechanistic insights from, logistically challenging in vivo trials. Although these models allow for the identification of candidate mechanistic pathways, many models involve supraphysiological dosages, nonphysiological conditions, or experimental changes relating to individual proteins or receptors, all of which limit translation to human trials. To overcome these drawbacks, the use of ex vivo human plasma and serum has been used in cellular models to investigate changes in myotube hypertrophy, cellular protein synthesis, anabolic and catabolic markers in response to differing age, disease states, and nutrient status. However, there are currently no concurrent guidelines outlining the optimal methodology for this model. This review discusses the key methodological considerations surrounding the use of ex vivo plasma and serum with a focus in application to skeletal muscle cell lines (i.e., C2C12, L6, and LHCN-M2) and human primary skeletal muscle cells (HSMCs) as a means to investigate molecular signaling in models of atrophy and hypertrophy, alongside future directions.

## Graphical abstract

**Figure:**
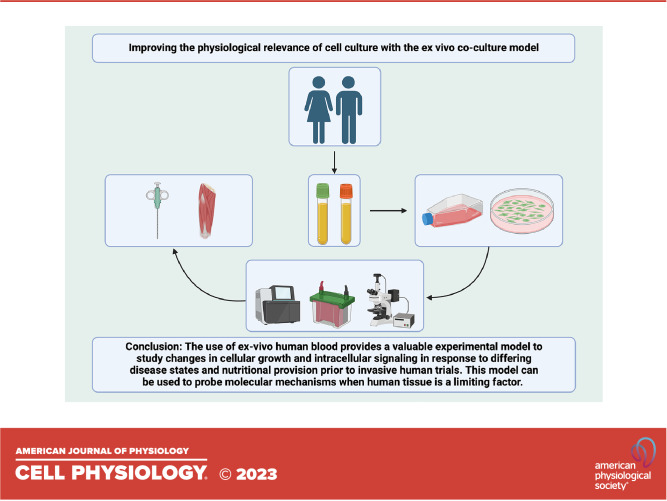

## INTRODUCTION

Over recent years the use of in vitro models in muscle physiology research has allowed for valuable investigations into the intracellular mechanisms of cellular growth and atrophy (1–3). Common in vitro models of skeletal muscle growth and atrophy include the use of immortalized cell lines obtained from mice (C2C12), rats (L6), and humans (LHCN-M2), induced through the incorporation of pharmacological treatments including insulin growth factor-1 (IGF-1), dexamethasone, and TNF- to investigate alterations in myoblast proliferation and muscle protein synthesis (MPS; 4–8). Although pharmacologically induced models of growth and atrophy highlight key targets of interest for further investigation, translation to in vivo human work may be limited. This may be partly due to basal culturing conditions. Immortalized skeletal muscle cell lines such as C2C12 are routinely cultured with Dulbecco’s modified Eagle’s medium (DMEM) supplemented with animal-derived serums i.e., fetal bovine serum (FBS) for proliferation and horse serum for differentiation (9). Although this combination provides the requirements to support optimal cell growth, a supraphysiological dose of nutrients are present in these media formulations (10). Therefore, traditional modes of cell culture create a microenvironment that lacks physiological relevance, which calls into question the validity of in vitro experimentation and the applicability of any findings to humans.

Undoubtedly, in vivo experiments with human participants provide the gold standard approach for the investigation of the mechanisms of muscle growth, atrophy, and potential health-promoting responses to nutraceutical and pharmacological compounds. However, due to ethical considerations and the logistically challenging nature of such studies (i.e., invasive, logistically complex and expense), there is an increasing demand for the development of a more physiologically relevant in vitro model to study muscle growth and atrophy that may better translate to the human model. To overcome these potential barriers, we (11–16) and others (17–20) have utilized ex vivo human serum or plasma to condition C2C12 and LHCN-M2 immortalized muscle cells and human primary skeletal muscle cells (HSMCs). Collectively, these studies have investigated a range of factors including changes in proliferation (19), myotube diameter (11, 13), anabolic (12, 15, 16) and catabolic signaling (18). Furthermore, such ex vivo approaches have allowed for valuable investigations into the effects of certain systemic environments e.g., aging, chronic disease, and nutrient quality (11, 13–16), thus providing a more physiological basis from which to study the molecular pathways influenced by differing cohorts and nutritional stimuli. Therefore, the purpose of this review is to provide an overview of the current understanding and methodology for the use of ex vivo human serum and plasma in an in vitro coculture model, specifically focused on the use of skeletal muscle cells, both from immortalized cell lines and primary cell cultures (i.e., C2C12 and HSMC, respectively). We will also discuss key methodological considerations and future directions to provide rationale and potential application of the model to move toward standardization of the use of this method by a wider range of researchers.

## HISTORICAL PERSPECTIVES AND CURRENT MODEL PROGRESSION

The ex vivo coculture model notably takes inspiration from previously established models of parabiosis (21). In 2005, Conboy et al. (22) created an experimental model in which two young and two old mice were paired to create a shared circulatory system through the formation of vascular anastomoses. This allowed for the exposure of differing systemic environments of young and older mice within a single system. During heterochronic parabiosis, exposure of older mice to a young, systemic environment led to significant improvements in notch signaling, proliferative and regenerative capacity of satellite cells (22). In contrast, exposure to an older systemic environment reduced regenerative capacity in younger mice (22). This model highlights the importance of the systemic environment, which contains the milieu of divergent hormonal, nutrient, and other humoral factors which regulate growth and atrophy, and thus provides a method that can allow for the investigation of age-related disease and longevity (21).

Similarly, the ex vivo coculture model utilizes ex vivo serum and plasma as a conditioning treatment, or complete replacement of animal serum in culture to investigate the response to exposure of differential systemic environments. Indeed, early studies which utilized human serum in coculture models aimed to compare the proliferation and differentiation capacity of various cell types, such as human bone marrow mesenchymal stem cells (hMSCs; 23–25) and stromal cells (26), in response to culturing with human serum in comparison with FBS (i.e., current standard conditions). The overarching aim of these early studies was to reduce the use of FBS in culture due to batch-to-batch variation and immunizing effects of xenogeneic proteins (23, 26). In response to these investigations, one of the first studies to investigate this approach showed an increased speed of proliferation in hMSCs incubated with human serum in comparison with standard protocols utilizing FBS (23). In support of these findings, Kobayashi et al. (24) found that human serum in replacement of FBS was sufficient to support cell proliferation of hMSC’s with an increase in cell viability over 6 days. Furthermore, recent work has identified no difference in population doubling times of human fibroblasts and adipose tissue-derived stem cells between human plasma and serum, highlighting the potential to utilize plasma, in addition to serum in coculture (27). Taken together this research highlights the viability of culturing stem cells in human plasma and serum and the potential to utilize these blood components to create more physiological culturing conditions.

More recently, ex vivo plasma and serum have been utilized in the coculture of skeletal muscle cells. These include immortalized cell lines such as C2C12 (11–16), L6 (28), LHCN-M2 (20), and HSMC (19) to investigate intracellular signaling in response to various treatments. One of the first studies to utilize the ex vivo model with muscle cells involved the culturing of HSMC from young and old donors (19). The authors found that serum from young and older donors induced no change in proliferation and differentiation in HSMC treated with 2% human serum between groups (19). This suggests that serum from differing age groups may not induce detectable changes in coculture models. In contrast, initial work in C2C12 skeletal muscle cells provided more promising results. In 2011 van Hees et al. (18) cocultured C2C12 skeletal muscle cells with 5% plasma collected from patients with septic shock to investigate markers of muscle protein breakdown (MPB) in C2C12s. The authors highlighted that plasma from patients with septic shock resulted in an increased gene expression of MuRF-1 and MAFbx, two proteolytic markers, and a reduction in myosin content (18). These findings contradict the findings of George et al. (19) suggesting that ex vivo blood components may be utilized to investigate changes in response to differential systemic environments in culture. Although the use of HSMC in combination with human serum may provide a gold standard approach, invasive procedures (i.e., muscle biopsies) are required to obtain muscle tissue for culture, thus increasing both study costs and recruitment challenges. Therefore, C2C12 and LHCN-M2 immortalized skeletal muscle cell lines may provide a suitable alternative model to study cellular signaling in combination with treatment of human serum or plasma. In more recent years, the model has been expanded to study the effects of various systemic environments including injury (18, 29), aging (11, 13, 19), disease (14, 17), nutrient sources (12, 15, 16), and exercise (20). An overview of studies using ex vivo plasma and serum in skeletal muscle cell types is presented in Table 1.

**Table 1.** Overview of different ex vivo protocols used in skeletal muscle cell models

Plasma/Serum	Duration and Dosage of Conditioning	Pooled vs. Individual	Author
*C2C12*			
LH Plasma	24 h, N/A	Pooled	Van Hees et al. (18)
Serum	24–96 h, 5%	Pooled	Corrick et al. (29)
LH Plasma	24–48 h, 5%	Individual	Kalampouka et al. (13)
Serum	4 h, 20%	Individual	Carson et al. (12)
Serum	4 h, 20%	Individual	Patel et al. (16)
Serum	4 h, 24 h, 10%	Individual	Allen et al. (11)
Serum	4 h, 20%	Individual	Lees et al. (15)
Serum	4 h, 24 h, 10%	Individual	Allen et al. (14)
*LHCN-M2*			
Serum	96 h, 0.5%	Pooled	Vitucci et al. (20)
*HSMC*			
Serum	120 h, 2%	Pooled + Individual	Catteau et al. (17)
Serum	46 h, 15% (proliferation), 144 h 2% (differentiation)	Individual	George et al. (19)
LH, lithium heparin			

### Development of the Ex Vivo Coculture Model in Muscle Cells

The development of the ex vivo coculture model in metabolic physiology has largely been driven by the desire to improve the translation of in vitro findings to in vivo human trials. As previously stated, the model has been utilized to create an in vitro model of aging, in our laboratories, and others (11, 13, 19). In contrast to the work by George et al. outlined earlier (19), Kalampouka et al. (13) investigated the influence of 5% human plasma from young and old donors on C2C12 skeletal muscle cells. We found that myoblasts treated with plasma from older donors displayed a lower ability to recover from injury induced via a scratch assay (13). In Addition, we found an increase in myotube diameter in C2C12 myotubes treated with 5% ex vivo plasma from young compared with old donors (13). To the best of our knowledge, this was the first study to highlight an aging-induced effect in a coculture skeletal muscle model. More recently, we have expanded upon these initial findings to show that C2C12 myotubes treated with 10% ex vivo human serum from young males led to an increase in myotube diameter compared with serum from older males (11). We also found an increase in MPS in response to in vitro 5 mM leucine treatment in C2C12s treated with 10% young serum, compared with treatment with fasted serum alone with no difference identified in old serum-treated cells (11). This highlights the utility of the model for investigating mechanisms of age-related anabolic resistance that have been well described in human in vivo experiments (30, 31). Taken together, the differences outlined herein may be a consequence of differing serum concentration (2% vs. 5%–10%) or cell type (i.e., immortalized mouse cell line vs. HSMC). Indeed, both C2C12s and HSMC display differential gene expression patterns (32). However, C2C12s have been shown to have similar amounts of myosin content and glycogen structure to primary HSMC (32). Thus, both C2C12 and HSMC have been suggested to be suitable for the investigation of myotube growth in response to stress (32).

In Addition, more physiologically relevant in vitro models are required to provide a platform in which the effectiveness of new nutraceutical and pharmacological treatments can be trialed. Currently, the ex vivo model has been used to investigate the anabolic properties of divergent nutrient sources after feeding (12, 15, 16). Initial work by Carson’s laboratory (12) examined whether the ex vivo model could be utilized to detect differences in anabolic signaling in C2C12’s conditioned with 20% human serum obtained at a fasted state or 60-min postprandial state in response to a whey protein bolus. We identified an increase in MPS in C2C12s conditioned with fed ex vivo human serum compared with fasted serum (12). This research was later expanded to investigate the sensitivity of the model to detect differences in different proteins of differing quality i.e., a whey protein isolate rich in essential amino acids (EAA’s) compared with nonessential amino acids (NEAA’s; 16). We highlighted an increase in MPS and mTOR-related signaling in C2C12s treated with 20% EAA fed serum compared with NEAA fed serum (16). Taken together, these studies highlight the capability of the model to investigate protein anabolism in response to proteins of differing qualities, a vital advancement due to the growing demand for alternative sustainable protein sources (33). As such, work by Lees et al. (15) used the ex vivo model to investigate differences in anabolic signaling in C2C12s after acute conditioning with 20% ex vivo human serum, obtained after the ingestion of fish-derived protein compared with whey protein isolate and NEAA in older adults. This study highlighted the anabolic potential of a novel sustainable fish-derived protein, thus providing a platform from which to base future in vivo human trials.

In addition to nutrient provision, exercise and physical activity supports muscle maintenance and adaptive remodeling and can improve health across the lifespan (34). Despite the importance of exercise, to the best of our knowledge, only one study has been conducted using the immortalized human skeletal muscle cell line (LHCN-M2) to investigate the influence of serum from different exercised subjects (20). In this study, serum was collected 8−10 h after a training session from participants who practiced volleyball, football, swimming, or body building for a minimum of 3 years and 180 min/wk (20). The authors found an increase in muscle-specific markers of early-stage (myogenin and creatine kinase activity) and late-stage differentiation (myosin heavy chain ) in cells treated with 0.5% exercised serum compared with untrained serum (20). Furthermore, differences were also detected between exercise modality, with serum from trained swimmers inducing a greater increase in myosin heavy chain in LHCN-M2 cells, compared with body building, football, and volleyball (20). This study suggests that serum from exercised individuals is a viable model, which can be used to study the effectiveness of exercise in culture. However, further research is warranted to assess the effect of serum obtained after resistance versus endurance exercise to investigate the potential drivers of tissue remodeling e.g., the role of extracellular vesicles (EVs) and provide an alternative approach to study exercise-mediated adaptations (35). This could provide an alternative model to study exercise in vitro as opposed to electrical pulse stimulation (EPS; 36). However, it is worth noting that this approach would remove the influence of mechanical stimuli, an influential factor associated with in vivo hypertrophy (34). Therefore, a more appropriate methodology may include the coculture of muscle cells in serum/plasma before EPS. Future research is required to determine the optimal model to study the effects of exercise in vitro.

Collectively, the ex vivo coculture model has been utilized to investigate divergent systemic environments in both skeletal muscle cell lines and primary skeletal muscle cells. Although a number of laboratories have adopted the use of human serum or plasma to condition culture medium, no consistent methodology is currently available for the inclusion of ex vivo human serum and/or plasma samples, thus advancements in this line of work have been limited. Therefore, the remainder of this article aims to discuss the practical considerations of the ex vivo coculture model.

## PRACTICAL CONSIDERATIONS FOR THE EX VIVO COCULTURE MODEL IN MUSCLE CELLS

### Systemic Considerations

**Figure 1.**
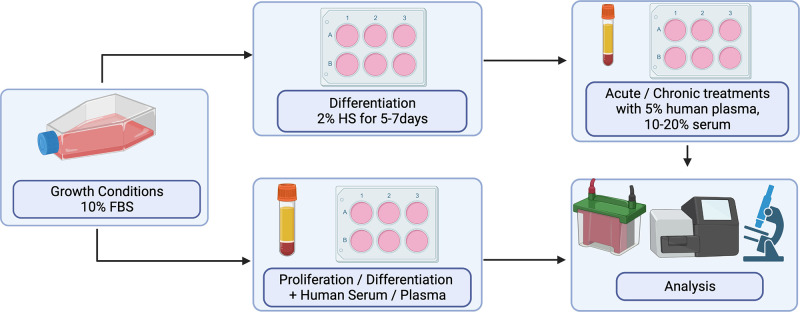
Schematic overview of the experimental set up of ex vivo cell culture. Skeletal muscle cells (cell lines or primary human cells) maintained under normal growth conditions should be plated for experiments. To assess intracellular signaling in myotubes, cells should be allowed to differentiate for 5–7 days before treatment with human plasma/serum. To assess markers of cell proliferation and differentiation, incubation with human plasma/serum should be completed once plated for experimentation. FBS, fetal bovine serum; HS, horse serum.

As highlighted, In the section *Development of the Ex Vivo Co-culture Model in Muscle Cells* both plasma and serum have been utilized to investigate the effects of differential systemic environments (Fig. 1). Numerous considerations are required for appropriate use of the ex vivo model, including the selection of blood components (i.e., plasma vs. serum) and dosage. Due to similarities in concentrations of a number of key chemical analytes such as glucose in plasma and serum, it is plausible to suggest that both could be utilized in culture interchangeably (37, 38). However, plasma has been shown to result in viability issues, likely a consequence of the presence of clotting factors and fibrinogen. Indeed, previous work has highlighted that plasma is less well tolerated by C2C12s compared with serum, 5% versus 10%–20%, respectively (11–13).

Furthermore, the anticoagulant used for plasma collection may influence cell viability. Previous work conducted in our laboratories has shown that ex vivo plasma collected in lithium heparin (LH) vacutainers was suitable for use in coculture models (13). In contrast, we observed that plasma collected in EDTA vacutainers led to media coagulation (unpublished data). These differential responses are likely due to the ability of LH vacutainers to inhibit coagulation through the activation of antithrombin and inhibition of thrombin (39). As a result, ex vivo plasma which is to be collected with the intended use being the ex vivo model should be collected in LH vacutainers, as opposed to EDTA vacutainers. However, due to an increased interest in cell culture models as a precursor to invasive human trials, we aimed to investigate whether plasma collected in EDTA vacutainers can be “rescued” for use in in vitro trials. We treated EDTA-plasma with heparin (∼2 units) for 30-min over ice and subsequently centrifuged plasma to ensure the removal of all heparin. We observed that heparinized EDTA-plasma prevented coagulation of culture media, without adversely affecting cell viability (unpublished data). Although this opens new possibilities for retrospectively collected plasma samples that were collected without the intention to utilize as an ex vivo treatment, we would stress that LH-collected plasma should be the current standard for plasma for ex vivo coculture.

### Experimental Setup: Sample Size, Dosage, and Timing

In addition to blood component type, the sample size, dosage, and timing of treatment must also be considered when designing an experiment that utilizes the ex vivo model. First, participant serum/plasma may be used for coculture experiments in two different ways, via an individualized approach (11, 13, 14), providing different biological replicates, or a pooled approach (17, 18, 29), involving the combination of samples from a group of participants. This approach will likely be influenced by a number of factors including the specific research question, sample availability, and ultimately the dosage and timing at which ex vivo samples will be applied. Although both approaches have strengths and limitations, consideration surrounding this allocation should be determined by the experimental aim. A participant-to-participant approach, where serum/plasma from each participant functions as a biological replicate is often utilized in “end-point” experiments after the course of differentiation. As such, previous work has used biological replicates to investigate cellular changes in MPS, anabolic and catabolic markers in response to 4-h treatments, and myotube structural changes in response to 24–48 h (11, 13, 14). Although this approach may result in an increased variability between biological replicates, it allows researchers to maintain a “biological” comparison within treatment groups. Therefore, an individualized approach may more closely replicate the in vivo results through participant-to-participant variability. These differences are likely a consequence of differences in circulating bioactives between participants. To determine the appropriate sample size for use, we conducted a power calculation based upon the effect sizes identified in previous research outlined within this review. We recommend that experiments should be conducted using 4–6 ex vivo samples (biological replicates) in triplicate utilizing three consecutive passage numbers to provide a technical replicate. Taken together, this approach offers a well-controlled and valuable model in which cellular mechanisms can be provided.

In contrast, pooled approaches have often been utilized for treatment over the course of proliferation or differentiation. Previous work which has used this approach has shown that a lower dosage of serum is required to maintain C2C12s or HSMC over the course of prolonged periods of time e.g., differentiation, similar to standard culturing conditions (i.e., 2% horse serum; 17, 19, 29). In 2015, Corrick et al. (29) showed that incubation with 5% pooled serum from burns patients induced a differential response in comparison with cells treated with 5% pooled serum from control patients. In contrast, more recent work by Catteau et al. (17) utilized a vol./vol. substitution of human serum (i.e., 2%) in replacement of horse serum throughout 5 days of differentiation. This approach highlighted myotube atrophy in cells treated with chronic obstructive pulmonary disease serum compared with healthy control serum. The differences in serum concentration between these two experiments may be reflective of the serum treatment periods (2–3 days vs. 5 days, respectively). These data suggest that a lower dosage (2%–5%) of human serum is required to investigate the changes induced throughout the differentiation period. Future work is warranted to investigate the suitability of ex vivo human plasma for prolonged experiments and to expand the usage of human serum over the course of proliferation and differentiation in further systemic environments i.e., aging. Furthermore, future research should aim to investigate the utility of coculturing cells with human serum/plasma at lower doses over the course of proliferation/differentiation before end-point treatment.

Dependent on the experimental aim, in an attempt to best represent the in vivo situation, researchers may look to maximize the dose of ex vivo human plasma/serum applied to the culture model. We have previously investigated the viability of C2C12 cells in high concentrations of ex vivo human serum and found concentrations as high as 50% were well tolerated for short periods (2–4 h), but lower concentrations (up to 20%) were well tolerated for up to 24 h (12). Higher concentrations may be of interest to researchers investigating acute responses, such as feeding, as this may best capture and expose cells in culture as close to the humoral milieu present in humans. Overall, the aim of this coculture model is to more closely mimic the interstitial environment that cells are exposed to in vivo, and it must be recognized that serum/plasma differs from interstitial fluids. Further research is required to determine the appropriate concentration of serum/plasma required to mimic the systemic environment in which in vivo muscle fibers would be exposed to, presenting a limitation of the ex vivo model.

### Experimental Controls

In addition to experimental conditions, an essential component of the experimental setup is the use of appropriate controls. Due to the application of ex vivo human serum/plasma, which often compares a number of different conditions, for example, healthy versus diseased (14, 17), young versus old (11, 13), fed versus fasted (12, 15, 16, 28), a number of different controls are required. First, in studies investigating the influence of aging, chronic disease, or acute injury a useful control measure may be the healthy control group. Similarly, in studies that aim to investigate the divergent effects of nutritional stimuli, basal fasted samples may act as a valuable control. In an exercise model, a resting nonexercise control may also be required. For both nutritional and exercise experiments, the fasting/resting control is important, particularly where biological replicates are used. We have previously observed significant interindividual differences in the bioactivity of fasting serum for example (16), therefore using each individual’s fasting serum as a control relative to the corresponding fed serum is imperative here. When utilized together, these baseline conditions act as vital controls to provide valuable insights into whether any changes found in response to incubation with differential systemic environments is due to a treatment effect.

Due to the nature of cross-species effects in response to treatment of an immortalized mouse cell line (C2C12s) with human serum, it is also important to investigate a control that is maintained under normal growth conditions throughout treatment e.g., 2% horse serum. Interestingly, we found that serum from young healthy control participants induced an increase in myotube diameter, in comparison with untreated control myotubes maintained in normal differentiation media (11). In contrast, 10% serum from old individuals induced a significant decrease in myotube diameter in comparison with both the young treated myotubes and control myotubes (11). Thus, it is plausible to suggest that the addition of human serum acts to “reset” cellular responsiveness due to the presence of a different composition of growth factors. As such, nontreated controls provide useful comparator to ensure a treatment effect is present.

Furthermore, acute experiments often utilize a serum starvation period, with or without an amino acid starvation period before treatment (11, 12). A starvation period is conducted before acute treatments to reduce MPS and anabolic signaling. Previous work from our laboratory has shown that 1 h of nutrient and serum starvation reduces MPS and the activation of mTOR, with no further suppression found after 4 h of starvation (12). Due to this additional methodological step, it is plausible to suggest that a serum-starved control condition should be utilized alongside those maintained under normal growth conditions. Therefore, experimental models should utilize a nonserum/plasma stimulated condition (i.e., FBS or horse serum only) alongside human serum/plasma treatments.

Alongside the use of appropriate controls, the most appropriate statistical test should also be considered. For example, when investigating differences between conditions such as disease or aging, a between-groups statistical test should be selected. Similarly, where a serum starvation or untreated control maintained under normal growth conditions is included in the analysis a between-groups statistical test should be selected. In contrast, where serum/plasma samples are obtained from the same participant at different time-points e.g., fasted versus fed samples, or involve the use of serum/plasma treatment with or without additional nutraceutical or pharmaceutical treatment within subjects statistical test should be utilized.

### Baseline Culturing Conditions

Alongside considerations around the use of ex vivo samples, baseline culturing conditions should also be acknowledged throughout the experimental design. Dependent on the experimental aim, the background or basal media that cells are cultured in may have an influence on experimental outcomes. For example, others have shown anabolic signaling in C2C12s is reduced when serum is removed (40) and we previously observed a blunting of the response to the addition of ex vivo human serum to C2C12s in the presence of DMEM and serum (unpublished data). This is likely due to the presence of nutrients (i.e., EAA’s) and growth factors at high concentrations in these media thus resulting in a saturation effect. Other nutrient factors, such as high or low glucose, should be considered depending on experimental aims.

### Considerations for Nonmuscle Cell Lines

Throughout this review, we have considered this protocol development in light of improving physiology relevance when using muscle cell lines. However, there is little practical reason why this method cannot be used with the majority of nonmuscle cell lines, except those inside the blood-brain barrier, such as in proliferation of cancer cell lines stimulated with plasma from pre- and postexercise (41, 42). It is worth noting that the ex vivo coculture model has already been applied to a number of nonmuscle cell lines such as adipocytes (27), liver cells (43), and neuronal cells (44).

## FUTURE DIRECTIONS AND NOVEL APPLICATIONS

**Figure 2.**
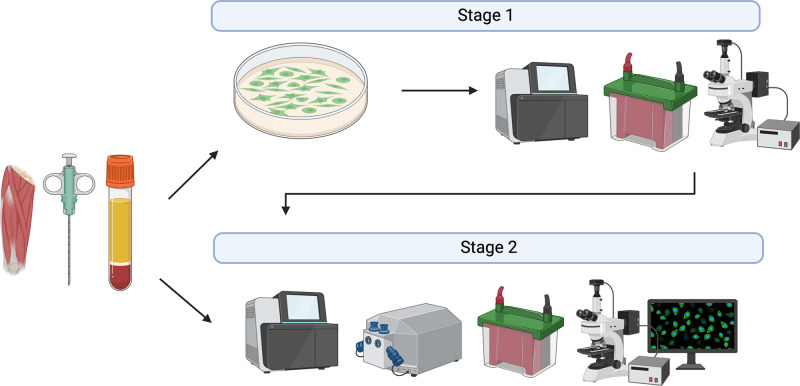
Recommended operational model for in vitro and in vivo work. We suggest that in vitro models should utilize human primary skeletal muscle cells or immortalized cell lines, e.g., C2C12s alongside human serum/plasma treatment. Results from in vitro studies may be used to inform in vivo human analysis. Further in vitro work may be conducted after human data collection to probe for further mechanistic data and the effectiveness of nutraceutical and pharmacological treatments to inform later in vivo work, based upon initial findings.

As highlighted throughout this review, the ex vivo model has the potential to study the intracellular mechanisms in response to a number of stimuli including differing disease states and nutritional status. This may be of particular importance when HSMCs are unavailable, and to provide valuable insights before logistically challenging in vivo trials (Fig. 2). However, future work should aim to investigate whether differences in signaling are present in response to ex vivo serum/plasma with HSMC versus C2C12 skeletal muscle cells to remove any potential “cross-species” effects and account for potential intrinsic muscular properties. Furthermore, it is worth noting that this model is not limited to use of human serum or plasma. Indeed, recent work has investigated the influence of hibernating bear serum on anabolic and catabolic markers alongside myotube growth (45). Finally, with the emerging role of EVs in cellular communication (46), including muscle responses to stimuli such as exercise (47), and observations that FBS contain functional EVs that directly inhibit C2C12 differentiation (48) one future direction of research could be EV-depleted plasma/serum alongside extracted and resuspended EVs.

## CONCLUSIONS

The application of ex vivo human serum/plasma to condition skeletal muscle myotubes provide a valuable experimental model to study changes in myotube morphology and metabolism in response to differing disease states and nutritional status-specific responses. Together, we have highlighted a clear framework for the use of ex vivo models in the future (Table 2). Briefly, we recommend that future researchers who wish to use this model should utilize serum or LH-collected plasma (5%–20%) from 4 to 6 volunteers (i.e., biological replicates) and repeat experiments using three technical replicates of consecutively passaged cells. For “end-point” studies, we recommend treatment periods of 4 h to investigate intracellular signaling and 24–48 h for the investigation of structural changes. Finally, to investigate changes over the course of cellular proliferation or differentiation we recommend that a lower dosage of plasma/serum be utilized (2%–5%) over a prolonged time frame (e.g., 72 h). Together, we hope that this review will improve the consistency and reliability of in vitro models in line with model progression.

**Table 2.** Ex Vivo Coculture Model Checklist

Blood Component	Serum/Lithium Heparin Plasma
Dosage and Timing	5%–20%, 4–48 h (end-point) 2%–5%, > 48 h (proliferation/differentiation)
Replicates	4–6 biological replicates, 3 technical replicates of consecutively passaged cells
Experimental Control	Untreated, basal conditions

## GRANTS

S.L.A and L.B are supported by the National Institute for Health Research (NIHR) Birmingham Biomedical Research Center (BRC-1215-20009). The views expressed as those of the authors and not necessarily those of the NIHR or the Department of Health and Social Care. B.P.C. is supported by The Department of Agriculture, Food and the Marine, B.T.E. is supported by The George Cayley Circle.

## DISCLOSURES

No conflicts of interest, financial or otherwise, are declared by the authors.

## AUTHOR CONTRIBUTIONS

S.L.A., B.T.E., B.P.C., and L.B. conceived and designed research; S.L.A. prepared figures; S.L.A., B.T.E., B.P.C., and L.B. drafted manuscript; S.L.A., B.T.E., B.P.C., and L.B. edited and revised manuscript; S.L.A., B.T.E., B.P.C., and L.B. approved final version of manuscript.
